# Effect of Nano-TiC Dispersed Particles and Electro-Codeposition Parameters on Morphology and Structure of Hybrid Ni/TiC Nanocomposite Layers

**DOI:** 10.3390/ma9040269

**Published:** 2016-04-06

**Authors:** Lidia Benea, Jean-Pierre Celis

**Affiliations:** 1 Competences Center: Interfaces-Tribocorrosion-Electrochemical Systems, Faculty of Engineering, Dunarea de Jos University of Galati, 47 Domneasca Street, Galati RO-800008, Romania; 2 Department of Metallurgy and Materials Engineering, Katholieke Universiteit Leuven, Kasteelpark Arenberg 44, Leuven B-3001, Belgium; Jean-Pierre.Celi@mtm.kuleuven.be

**Keywords:** nanostructured, nanocomposite, hybrid layer, nano-TiC dispersed phase, electron microscopy, X-ray diffraction, nanoindentation, roughness

## Abstract

This research work describes the effect of dispersed titanium carbide (TiC) nanoparticles into nickel plating bath on Ni/TiC nanostructured composite layers obtained by electro-codeposition. The surface morphology of Ni/TiC nanostructured composite layers was characterized by scanning electron microscopy (SEM). The composition of coatings and the incorporation percentage of TiC nanoparticles into Ni matrix were studied and estimated by using energy dispersive X-ray analysis (EDX). X-ray diffractometer (XRD) has been applied in order to investigate the phase structure as well as the corresponding relative texture coefficients of the composite layers. The results show that the concentration of nano-TiC particles added in the nickel electrolyte affects the inclusion percentage of TiC into Ni/TiC nano strucured layers, as well as the corresponding morphology, relative texture coefficients and thickness indicating an increasing tendency with the increasing concentration of nano-TiC concentration. By increasing the amount of TiC nanoparticles in the electrolyte, their incorporation into nickel matrix also increases. The hybrid Ni/nano-TiC composite layers obtained revealed a higher roughness and higher hardness; therefore, these layers are promising superhydrophobic surfaces for special application and could be more resistant to wear than the pure Ni layers.

## 1. Introduction

Research studies have shown that the use of nanotechnology in coatings has significantly increased in recent years. Thus, composite and hybrid materials with new structures and special functions have increasingly come under a spotlight [1,2,3,4,5,6].

Composites are used due to their structural, electrical, thermal and tribological properties. Composite materials are usually optimized to achieve a particular balance of properties for a specific range of applications [2]. For example, aircraft engineers are looking for structural materials that have low density, are more powerful, rigid, resistant to abrasion and impact, and not easy to corrode [3].

Many of the modern technologies require materials with unusual combinations of properties that can not be met by conventional metal alloys, ceramics and polymers. Nanocomposite development provides for new technologies and business opportunities in many sectors of aerospace, automobile, electronics and biotechnology industries [4]. The effect of bath ionic strength on adhesion and tribological properties of pure nickel and Ni-based nanocomposite coatings is studied [5]. Ni-based nanocomposite coatings (Ni-Al_2_O_3_, Ni-SiC and Ni-ZrO_2_) were also produced on steel substrate and evaluated for industrial performance of wear resistance, corrosion resistance, adhesion strength and wettability behavior [6]. Nanocrystalline Ni-W alloy metallic matrix reinforced with ZrO_2_ particles (average size of 50 nm) with enhanced functional properties have been developed [7].

Titanium carbide (TiC) is widely used as a reinforcing particle to produce metal matrix composites due to its hardness, chemical inertia, high melting point and stability. Composites comprising titanium carbide metal matrix can be characterized by good wear resistance with a relatively low coefficient of friction [8,9].

The literature specifies various preparation methods such as electrodeposition [10,11] physical vapor deposition, chemical vapor deposition, thermal, plasma spraying [12], *etc.*—methods that have been investigated to create composite materials.

Electrodeposition offers advantages over the abovementioned technologies, as it requires simple equipment and operating conditions and can be used to be deposited on irregular geometries, is low cost, has good reproducibility, a homogeneous distribution of particles and, last but not least, reduced waste [13]. The structure and properties of the composite layers obtained electrochemically depend not only on the concentration, size distribution and nature of the particles incorporated but also on other parameters such as current density, particle concentration, stirring, temperature, pH and electrolyte bath composition. The concentration of particles in the electrolyte bath, current density and bath stirring seem to be the most important parameters [14]. Superhydrophobic surfaces have been reported in the literature over the past decade using electrodeposition as a synthesis technique [15].

Surfaces of pure nickel (Ni) show low values of mechanical properties such as hardness and wear resistance [11]. In order to improve the surface properties, research is carried out on Ni electrodeposition with various types of reinforcing particles. Titanium carbide (TiC) is widely used as a reinforcing particle to produce metal matrix composites due to its hardness, chemical inertia, high melting point, good electrical conductivity, stability and mechanical properties [14,16,17].

This research work aims to investigate the effects of operating parameters such as current density, electrodeposition time, concentration of TiC nanoparticles (50 nm average size) in the deposition bath on their inclusion into the nickel matrix, on the surface morphology, structure and the thickness of the hybrid nanostructured layer thus obtained.

## 2. Experimental

### 2.1. Preparation of Ni/TiC Nanostructured Layers

**Figure 1:**
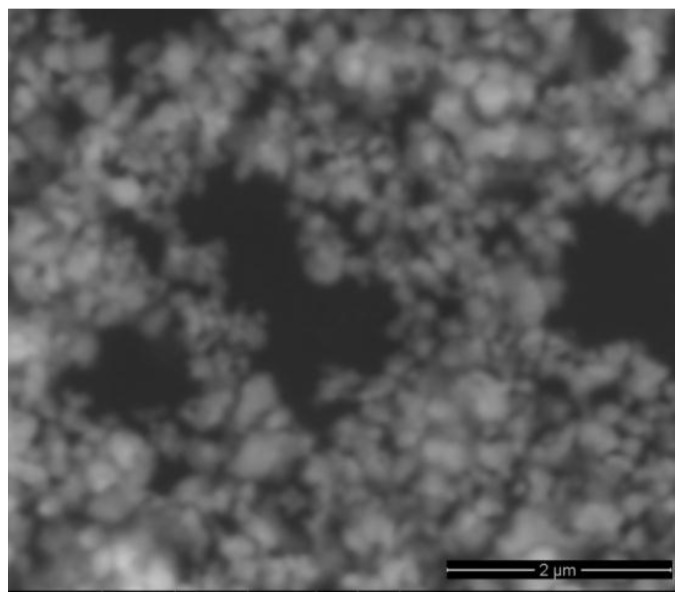
SEM surface morphology of TiC nanoparticles.

Ni/TiC nanostructured layers were electrolytically deposited from a standard nickel Watts plating bath to which TiC dispersed nanoparticles with different concentrations were added (mean diameter size of 50 nm, supplied by Hefei Kaier Nanometer Energy & Technology Co., Ltd., Hefei, Anhui, China. The SEM surface morphology of TiC nanoparticles before introducing them into nickel electrolyte is shown in Figure 1. The concentrations of TiC dispersed phase added in the electrolyte were 10 g·L^−1^, 15 g·L^−1^ and 20 g·L^−1^.

To obtain electro-codeposited coatings, a 304 stainless steel support was used as the cathode (WE). Before electrodeposition, the steel substrates were cleaned in alkaline cleaning solution followed by an acid etch process. A pure nickel plate and a saturated calomel electrode (SCE) (Hg/Hg_2_Cl_2_, KCl, E = +244 mV *vs.* normal hydrogen electrode, NHE) were used as a counter electrode and reference electrode (RE), respectively.

**Figure 2:**
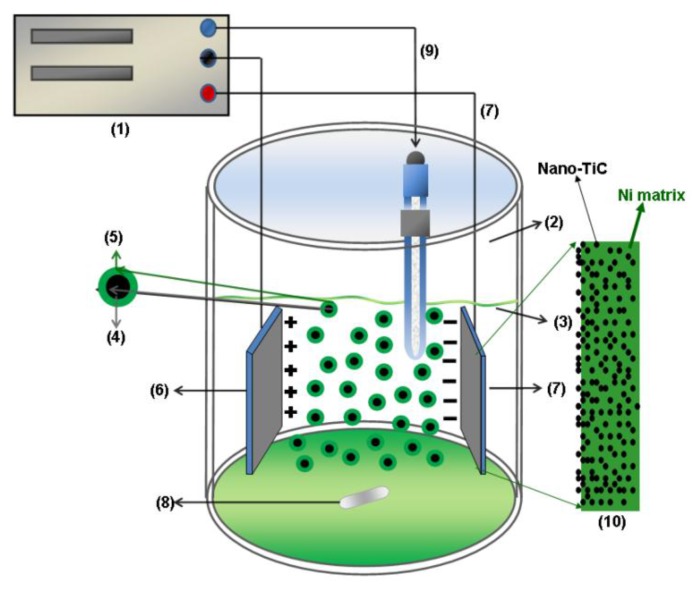
Schematic presentation of the electrolytic cell to obtain Ni/TiC nanostructured composite layers: (**1**) potentiostat/galvanostat; (**2**) electrochemical cell; (**3**) electrolyte; (**4**) nano-TiC (50 nm) dispersed particles; (**5**) ionic shell adsorbed around TiC particles; (**6**) anode (CE); (**7**) cathode (WE); (**8**) magnetic stirrer; (**9**) reference electrode (RE); (**10**) schematic representation of electrodeposited hybrid composite layer.

Electrodeposition took place in the electrolytic bath at 45 ± 1 °C, having a pH of 4.04. A double walled electrochemical cell containing the three electrodes (WE, counter electrode—CE, RE) was used to keep the desired temperature. The electrodes were positioned vertically and parallel having the same distance for each measurement. In order to ensure the uniform dispersion of nanoparticles, the electrolytic bath was stirred using a magnetic stirrer. From many experiments, a stirring rate value of 400 rpm resulted in being the best one to maintain the nanoparticles in homogenous dispersion in all electrolytes. Schematic presentation of the electrolytic cell is shown in Figure 2. In order to maintain a constant content of nickel ions into the nickel plating bath a nickel plate was used as counter electrode or anode (CE).

The electrolytes contain nickel salts dissolved, which form nickel ions. After immersion of nano sized TiC particles into nickel electrolyte, a dispersion of particles surrounded by ions adsorbed from solution is formed. Only positively charged particles (surrounded by nickel ions or even hydrated hydrogen ions) are supposed to migrate to the cathode together with nickel ions to form a further nickel matrix with nano TiC particles included as Ni/TiC nanostructured layers, following the equation:

(1) $${\left(Ni^{2+}\right)}_{ads}(nano-TiC)\overset{+2e^{-}}{\to }Ni/nano-TiC\;\text{nanostructured hybrid composite layer}$$

In a recently published review [18], mathematical modelling of composite plating is discussed, and the model involving electrophoresis of particles is said to still be valuable and widely used to approximately describe many composite plated systems. There are two ways which a particle will respond to the external field. If the particle is charged, it will migrate in an electric field to the electrode of opposite charge, following the principle of electrophoresis. Therefore, only the positive charged particles will migrate to the cathode.

The deposition was carried out at different current densities: 40 mA/cm^2^, 60 mA/cm^2^ and 72 mA/cm^2^ for different deposition times of 15 min and 30 min Pure Ni deposits were also produced under the same experimental conditions for comparison.

Electrodeposition of the layers in the absence and presence of the dispersed nano-TiC phase was performed by using a potentiostat/galvanostat. The following nanostructured layer systems were obtained and comparatively characterized: Ni/nano-TiC (0 g·L^−1^), also called pure Ni layer, Ni/nano-TiC (10 g·L^−1^), Ni/nano-TiC (15 g·L^−1^) and Ni/nano-TiC (20 g·L^−1^).

### 2.2. Characterization of the Electrodeposited Layers

The surface morphology and the composition of the nanostructured Ni/TiC layers obtained electrochemically were characterized by scanning electron microscopy (SEM-Philips XL 30 FEG, Leuven, Belgium) with energy dispersive X-ray spectroscopy (EDX). In addition, the incorporation percentage of TiC nanoparticles in the Ni matrix was estimated by EDX.

The X-ray diffraction analyses were performed using a Seifert 3003 T diffractometer with a Cu Kα radiation (GE Inspection Technologies, Leuven, Belgium), operated at 40 kV and 40 mA. The diffractograms were recorded with a step of 0.02°, in 2*θ* geometry, ranging from 10° to 100° and measuring time 2 s per step. The XRD study was carried out on all the coated samples to identify the presence of TiC dispersed phase incorporated into nickel matrix and to compare the texture of the deposited coatings.

Coating thickness was measured by weighting the samples, before and after electrodeposition. In order to confirm the coating thicknesses calculated by weighting, the real thicknesses were also measured by SEM on a cross section of the samples. Therefore, the samples were embedded in epoxy resin, cut in a cross section, and then successively polished with abrasive paper with various grit and diamond paste (1 and 3 μm size), cleaned in an ultrasonic ethanol bath and dried using dry, cold air. Then, the samples were coated with a very thin layer of gold.

The surface roughnesses of the pure nickel and Ni/TiC hybrid nanostructured layers were determined by white light interferometry analysis using a Wyco NT3300 optical profilometer (Veeco, Leuven, Belgium) with Vision (version 2.210) software (Veeco, Leuven, Belgium).

The pure Ni layer and Ni/TiC hybrid nanostructured layers were tested by nano indentation testers (CSM-instruments equipped with a Berkovich diamond indenter, CSM Instruments, Leuven, Belgium) in order to extract elastic modulus and hardness of the specimen material from load-penetration depth measurements. The applied loading force was 10 mN, with a 5 s pause time after each measurement.

## 3. Results and Discussion

### 3.1. SEM Surface Morphology of Ni/Nano-TiC Hybrid Nanocomposite Layers

Analysis of electrodeposited layers by scanning electron microscopy revealed differences in surface morphology of Ni/TiC nanostructured layers compared to pure Ni electrodeposited layer obtained at same parameters. The surface morphologies of the obtained layers are significantly different and depend on the current density used, electrodeposition time and the amount of TiC dispersed nanoparticles added to the plating bath.

**Figure 3:**
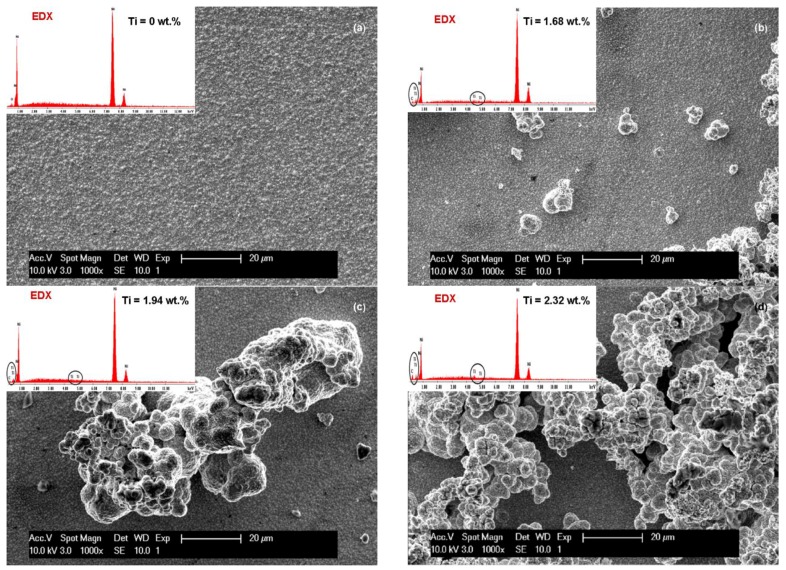
SEM surface morphology and EDX compozition for electrodeposited layers: (**a**) Ni/nano-TiC (0 g·L^−1^); (**b**) Ni/nano-TiC (10 g·L^−1^); (**c**) Ni/nano-TiC (15 g·L^−1^); and (**d**) Ni/nano-TiC (20 g·L^−1^) obtained at current density of 40 mA/cm^2^ and deposition time of 15 min. The labelled element in EDX spectrum is titanium (Ti), which confirm the inclusion of TiC particles.

**Figure 4:**
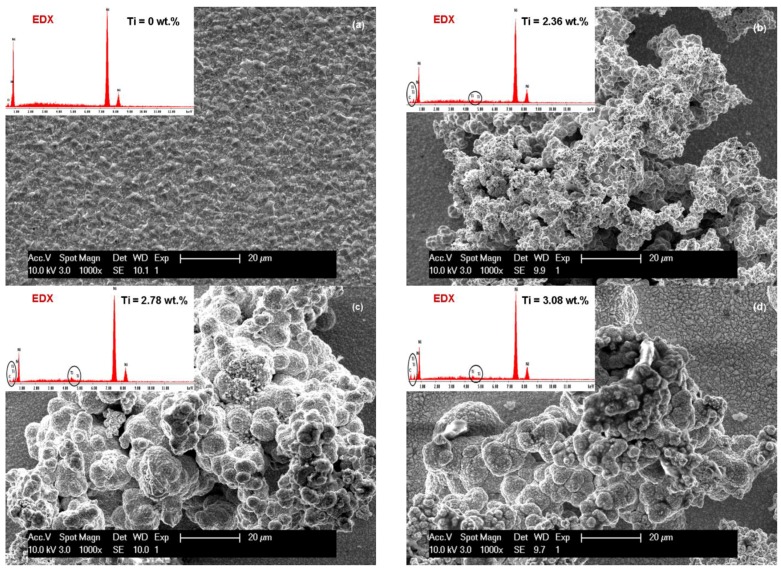
SEM surface morphology and EDX compozition for electrodeposited layers: (**a**) Ni/nano-TiC (0 g·L^−1^); (**b**) Ni/nano-TiC (10 g·L^−1^); (**c**) Ni/nano-TiC (15 g·L^−1^); and (**d**) Ni/nano-TiC (20 g·L^−1^) obtained at current density of 40 mA/cm^2^ and deposition time of 30 min. The labelled element in EDX spectrum is titanium (Ti), which confirm the inclusion of TiC particles.

The influence of current density (40 mA/cm^2^ and 72 mA/cm^2^) on the SEM surface morphology of the pure Ni and Ni/TiC layers containing different concentrations of TiC nanoparticles is depicted in Figure 3a–d and Figure 4a–d. It is evident that embedding TiC nanoparticles into the nickel matrix provokes changes in the morphology of pure nickel matrix. A regular pyramidal structure, as shown in Figure 3a and Figure 4a, is observed on the surface of the pure nickel layers, with clear edges of the grains. The SEM surface micrographs of pure Ni deposits (Figure 3a and Figure 4a) showed also that by increasing the current density, the crystallite size of the pure nickel layer becomes bigger, this behavior being confirmed further by XRD analysis. This observation is consistent with the results reported by Thiemig *et al.* [19] and Ebrahimi *et al.* [20] for pure Ni electrodeposited layers. Ebrahimi *et al.* [20] have suggested that this behavior is associated with a drop in the electrodeposition efficiency and the evolution of more hydrogen at the cathode/electrolyte interface. According to them [20], the changes in the surface energy and growth mechanisms in the presence of hydrogen are suggested as being responsible for the increase in the crystallite size.

With the first addition of TiC nanoparticles (10 g·L^−1^) in the nickel plating bath (Figure 3b and Figure 4b), the boarders of the grains become blurred and the mean grain size is diminished compared to pure Ni layers at the same current density. Further increase in nanoparticles amount to 15 g·L^−1^ in the electrolyte (Figure 3c and Figure 4c) leads to a characteristic cauliflower surface morphology. This observation was also reported earlier by Spanou *et al.* [21] for Ni/nano-TiO_2_ composite electrodeposits. TiC nanoparticles show a distinct tendency to form spherical agglomerates uniformly distributed over the whole surface of the coating. This agglomeration tendency increases with increasing the concentration of the TiC dispersed phase added to the nickel electrolyte and also with increasing the current density.

The effect of electrodeposition time on the SEM surface morphology of the Ni/nano-TiC (20 g·L^−1^) layers is shown in Figure 3d and Figure 4d. From these figures, it can be seen that the agglomeration effect is more pronounced for a higher deposition time, and, therefore, the cauliflower surface morphology is more obvious.

It can be seen that, with the addition of TiC nanoparticles, the nanocomposite layer surface changes and the grains become smaller compared with the pure Ni layer surface.

From Figure 3b, it can also be seen that the surface layer of Ni/TiC nanocomposite (10 g·L^−1^) is more homogeneous compared to the surfaces of other nanocomposites with 15 g·L^−1^ and 20 g·L^−1^ TiC obtained at electrochemical current density of 40 mA/cm^2^ over 15 min according to the SEM images.

From the same SEM images (Figure 3), the reduction of the grain size of crystallites due to the presence of TiC nanoparticles is also observed. On the nanocomposite layer surfaces, a nodular surface structure is formed, with observation confirmed by other researchers [22] for cerium oxide codeposited with nickel matrix.

By increasing the time for electrodeposition (Figure 4) and concentration of TiC nanoparticles an agglomerated cluster was formed on the surface of the nano-composite layers, but the presence of nanoparticles in the EDX spectra can be noticed.

**Figure 5:**
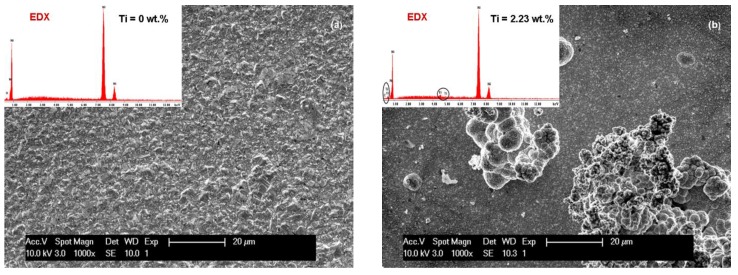

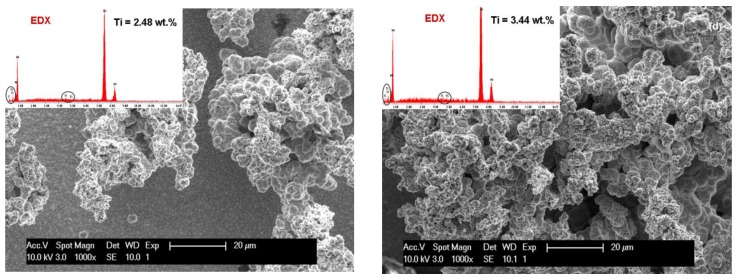
SEM surface morphology and EDX compozition for electrodeposited layers: (**a**) Ni/nano-TiC (0 g·L^−1^); (**b**) Ni/nano-TiC (10 g·L^−1^); (**c**) Ni/nano-TiC (15 g·L^−1^) and (**d**) Ni/nano-TiC (20 g·L^−1^) obtained at current density of 72 mA/cm^2^ and deposition time of 15 min. The labelled element in EDX spectrum is titanium (Ti), which confirm the inclusion of TiC particles.

**Figure 6:**
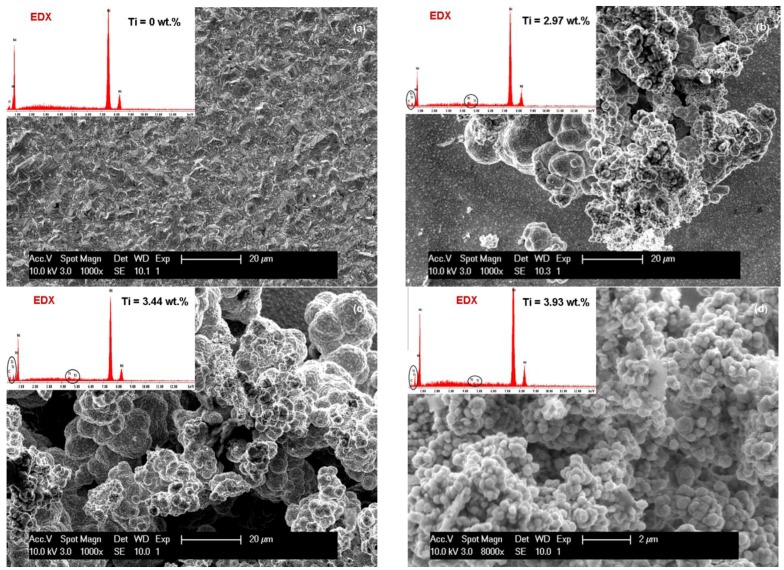
SEM surface morphology and EDX compozition for electrodeposited layers: (**a**) Ni/nano-TiC (0 g·L^−1^); (**b**) Ni/nano-TiC (10 g·L^−1^); (**c**) Ni/nano-TiC (15 g·L^−1^); and (**d**) Ni/nano-TiC (20 g·L^−1^) obtained at current density of 72 mA/cm^2^ and deposition time of 30 min. The labelled element in EDX spectrum is titanium (Ti), which confirm the inclusion of TiC particles.

Figure 5 and Figure 6 show the SEM surface morphologies and compositional analysis by EDX spectra of the pure nickel layers (Ni/nano-TiC (0 g·L^−1^) and the Ni/nano-TiC composite layers at three concentrations (10, 15, 20 g·L^−1^) obtained at the electrochemical current density of 72 mA/cm^2^ during the co-deposition time of 15 min (Figure 5) and 30 min (Figure 6).

By using a higher current density of 72 mA/cm^2^, an increased amount of nickel crystallites are formed on the surface layer of nickel matrix. These morphological changes of the layers of pure Ni at about the same current density have been reported by other authors [23] for electro-codeposition of alumina into nickel matrix.

By increasing the current density to 72 mA/cm^2^, and thus the time of codeposition, the surface morphologies’ nano-composite layers become more like clusters and agglomeration nano-composite layers as shown in Figure 5 and Figure 6. The reason could be that the TiC nanoparticles are good conductors, and the resulting electric field is stronger around clusters than elsewhere due to increased current density. This observation has been reported by other authors but by using other composites such as Ni-carbon black (CB) and Ni-carbon nanotubes (CNT) [24,25].

### 3.2. Nano TiC Particles Incorporation into Nickel Matrix

**Figure 7:**
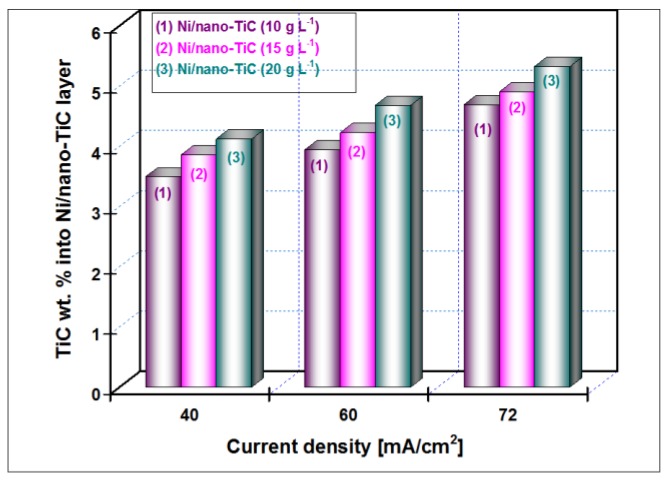
The degree of nano-TiC inclusion into nickel matrix depending on the current density at the time of codeposition of 15 min and different concentration of TiC nanoparticles added to the nickel electrolyte.

The EDX analyses were collected from the entire scan area of the samples in order to examine their elemental composition. The incorporation percentage of TiC into Ni matrix was determined by transformation of Ti weight percentage (wt. %) in molecular mass of titanium carbide. In the TiC compound, the Ti element has a value of 79.941 wt. %. The weight percentage for Ti element included in the Ni matrix (Figure 3, Figure 4, Figure 5 and Figure 6) as determined by EDX analysis is presented in Figure 7 for the current densities of 40 mA/cm^2^, 60 mA/cm^2^ and 72 mA/cm^2^ at a codeposition time of 15 min.

**Figure 8:**
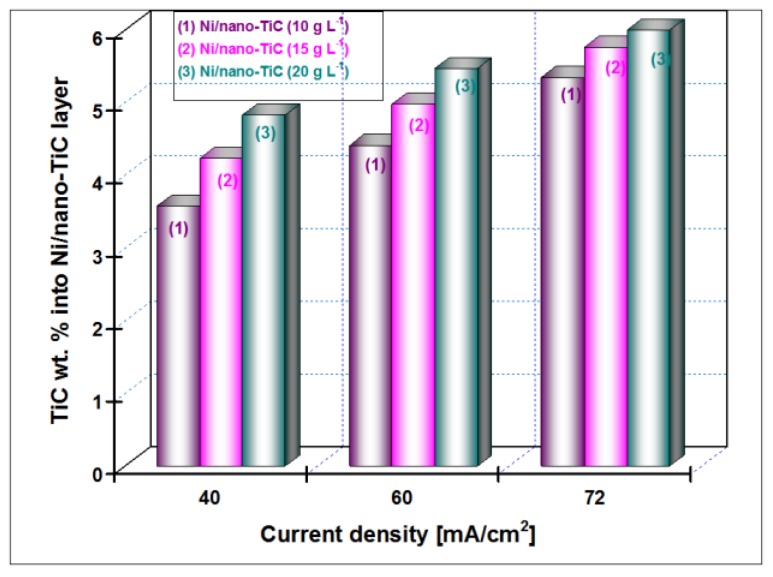
The degree of nano-TiC inclusion into nickel matrix depending on the current density at the time of codeposition of 30 min and different concentration of TiC nanoparticles added to the nickel electrolyte.

The weight percentage (wt. %) of the TiC nanoparticles incorporation into nickel matrix for all examined nanostructured layers (10, 15 and 20 g·L^−1^ concentrations of TiC), with the current density (40 mA/cm^2^, 60 mA/cm^2^ and 72 mA/cm^2^) at a codeposition time of 30 min., as was calculated from compositional EDX analysis is shown in Figure 8.

It is indicated that the wt. % of TiC nanoparticles embedded into Ni/TiC nanostructured layers is dependent on the TiC dispersed nanoparticles concentration in the nickel plating bath and the applied current density. The wt. % of TiC into Ni/nano-TiC layer increases with increasing of TiC nanoparticles concentration in the electrolyte at the same current density. A similar behavior was reported by studies related to the electro-codeposition of TiO_2_ nanoparticles with different concentrations into nickel matrix [26]. It may also be noted that the wt. % of TiC into nanostructured Ni/TiC electrodeposited layer increases with increasing the current density for each system separately—see Figure 7 and Figure 8.

The variation of wt. % of the TiC nanoparticles incorporation into the nickel matrix for all examined Ni/TiC nanostructured composite layers (10, 15 and 20 g·L^−1^ concentration of TiC in the electrolyte), as a function of codeposition time (15 min and 30 min) and a constant current density of 40 mA/cm^2^, as a result of EDX analysis and shown in Figure 7 and Figure 8, conclude that an increase of the codeposition TiC wt. % nanoparticles into hybrid nanostructured composite layer is possible by increasing their concentration added to the nickel electrolyte and by increasing the codeposition time.

### 3.3. Current Efficiency During Electro-Codeposition

The electrodeposition process is frequently been evaluated by using one parameter to achieve an overall understanding of the three-dimensional electrodes that is current efficiency. Current efficiency (η) is the ratio of the mass of metal deposited at a given time to the mass that would be deposited if all of electric current were used in the electrolytic process (Equation (2)):

(2) $$\eta =\frac{zF}{MI}\frac{\Delta m}{\Delta t}$$

where *z* is the number of electrons involved in the electrochemical reaction (for nickel, *z* = 2); *F* is the Faraday constant (96,487 Coulomb/mol); *M* is atomic weight (58.69 g·mol^−1^ for the nickel); *m* is the mass electroprocessed during the time interval Δ*t* (g); and *I* is the electric current (A).

**Figure 9:**
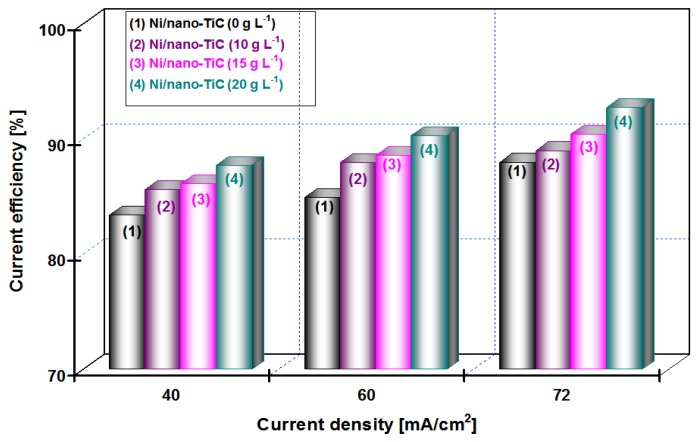
Current efficiency *versus* applied current densities at different concentrations of nano TiC particles added to nickel electrolyte at deposition time of 15 min.

**Figure 10:**
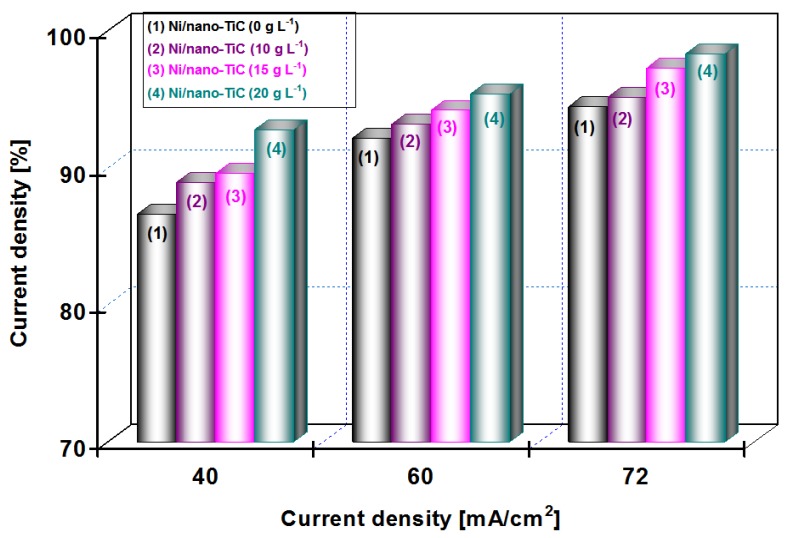
Current efficiency *versus* applied current densities at different concentrations of nano TiC particles added to nickel electrolyte at deposition time of 30 min.

The influence of applied current densities of 40, 60 and 72 mA/cm^2^ on the efficiency of the deposited layers (current efficiency), η (%) for the pure Ni and the nanocomposite Ni/nano-TiC layers obtained at different concentrations of nanoparticles (10, 15 and 20 g·L^−1^) and the co-deposition time (15 min and 30 min), is shown in Figure 9 and Figure 10.

From Figure 9 at a codeposition time of 15 min, it can be seen that the current efficiency of Ni/nano-TiC hybrid nanocomposite layer is about 92% at a current density of 72 mA/cm^2^. From Figure 10, at a codeposition time of 30 min, the current efficiency of Ni/nano-TiC nanocomposite layer reached about 98% at the same current density.

The higher current efficiency obtained during electro-codeposition of nano-TiC particles with nickel could be explained by embedding these nanoparticles into a nickel matrix during the electroplating process.

The results presented in this chapter concludes the beneficial effect of TiC nanoparticles in increasing the current efficiency at electro-codeposition of Ni/TiC hybrid nanocomposites compared with pure nickel layers.

### 3.4. X-ray Diffraction Patterns of Ni/TiC Hybrid Nanocomposite Layers

**Figure 11:**
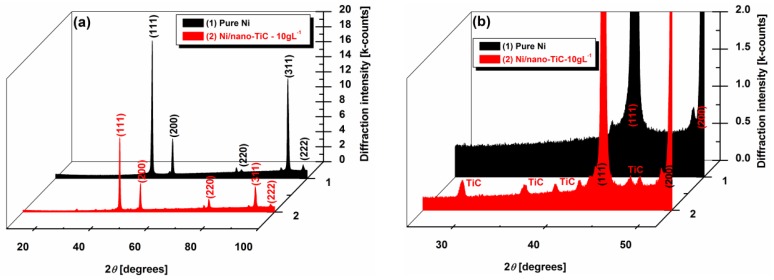
XRD patterns of: (1) pure Ni and (2) Ni/nano-TiC (10 g·L^−1^) functional coatings produced under same experimental conditions, at (**a**) a full magnification; (**b**) a zoom in the scale range to see diffraction peaks of TiC particles embedded into nickel matrix.

Figure 11a,b show the X-ray diffraction analysis at different scale ranges of the pure Ni and the Ni/nano-TiC functional nanocomposite coatings with a concentration of 10 g·L^−1^. The diffractograms presented in Figure 5a are characterized by the (111), (200), (220), (311) and (222) diffraction peaks. The same X-ray diffraction patterns of the electrodeposited nanocrystalline nickel coatings were also obtained by other researchers [19,27]. The presence of TiC nanoparticles in the composite is also noticed in XRD analysis, as can be seen in Figure 11b, which represents the enlarged region of Figure 11a, with diffraction peaks of TiC particles embedded into the nickel matrix.

It can be seen that the pure Ni coating has the most intense peaks with 2θ angles of 44.6°, 51.9° and 93°. These peaks are attributed to the crystalline planes (111), (200) and (311), respectively, as was also reported by other researchers [19,28].

The maximum values of intensities corresponding to the planes (111), (200) and (311) for pure nickel and Ni/nano-TiC coatings deposited from electrolytes containing 10 g·L^−1^ TiC are given in Table 1, as they resulted from Figure 11a.

The intensity of the diffraction peaks of the nickel in the Ni/nano-TiC nanocomposite functional coating is observed to be lower (Table 1), and the peak width is broader compared with that of the pure nickel coating (Figure 11a). This is attributed to the decrease in the crystallite size of the Ni/nano-TiC coating by the addition of TiC particles into the plating bath. A similar effect of particle addition on grain size has also been reported earlier by Arghavanian *et al.* [29] for Ni-ZrO_2_ composite coating (ZrO_2_ powder with a particle size of 1–5 μm) compared with pure nickel coating, and by Vaezi *et al.* [30] for Ni-SiC nano-composite (SiC nano-particulates of 50 nm mean diameter) as compared with nickel coating.

The relative texture coefficients, RTC_111_, RTC_200_ and RTC_311_, corresponding to (111), (200) and (311) crystallographic planes, were calculated according to Equation (3) and are given in Table 2.

(3) $$RTC_{hkl}=\frac{I_{hkl}/I_{hkl}^{0}}{\sum_{1}^{5}I_{hkl}/I_{hkl}^{0}}\times 100\%$$

where *I_hkl_* are the relative intensities of the (*hkl*) reflections, Ʃ*I_hkl_* is the sum of all intensities, in our case (111), (200), (220), (311) and (222). $I_{hkl}^{0}$ are the relative intensities of a randomly oriented nickel powder sample, from JCPDS No. 4-850 (Joint Committee on Powder Diffraction Standards) [19,28].

From the relative texture coefficient (*RTC*) value given in Table 2, it has been observed that the pure Ni coating from the particle free bath shows more texturing along the (311) plane, resulting in preferred orientation along the (311) plane. The addition of TiC nanoparticles into the bath modifies the preferred orientation of the pure Ni coating along the (311) crystallographic plane, reducing the *RTC* value at 28.73 (about half), compared with that of the pure Ni coating. The TiC nanoparticle incorporation caused a loss of texture, indicated by the decreasing of *RTC_311_* and the increasing of *RTC_111_* and *RTC_200_* values (Table 2).

These observed changes in *RTC* are sufficient to prove that the wear properties of the Ni/TiC nanocomposite coatings will be improved, because is know that, in the crystallographic plane (111), adjacent atomic layers can slide over each other with minimum friction. This last statement is supported by the fact that an (111) oriented deposit exhibits superior wear performance, and an increasing of the relative texture coefficient from the (111) plane means that the coating becomes stronger [31,32].

### 3.5. Thicknesses of Ni/Nano-TiC Hybrid Nanostructured Layers

**Figure 12:**
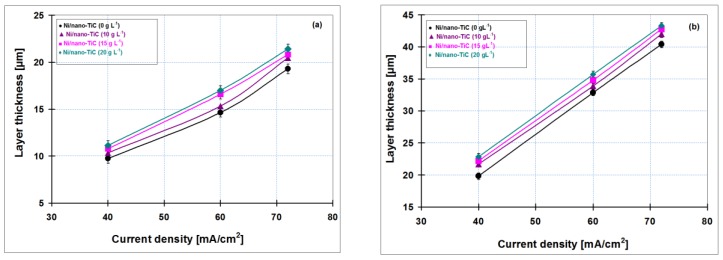
Layer thickness variation depending on the current density by weighting and SEM in cross section of layers of pure Ni and Ni/nano-TiC (10, 15 and 20 g·L^−1^) at the codeposition time of: (**a**) 15 min; and (**b**) 30 min.

Figure 12 shows the correlation between the thickness obtained by weighting and SEM cross section as the average of three measurements *versus* current density of codeposited layers for all nanoparticle concentrations of titanium carbide added to nickel electrolyte (10, 15 and 20 g·L^−1^) compared to pure nickel layer obtained at 15 min (Figure 12a) and 30 min (Figure 12b).

Figure 12a,b indicate that the thickness of electrodeposited layers increases with increasing current densities; this upward trend was obtained for all types of codeposited layers. In addition, the layer thickness increases with increasing concentration of TiC nanoparticles in the nickel electrolyte solution. By adding 20 g·L^−1^ TiC nanoparticles in the nickel plating bath, the Ni/nano-TiC layer thicknesses are higher as compared with pure nickel layer and the others composite systems, confirming the incorporation of titanium carbide nanoparticles into metallic matrix. Due to increasing of electrodeposition time, an increase in coating thickness has also been observed.

**Figure 13:**
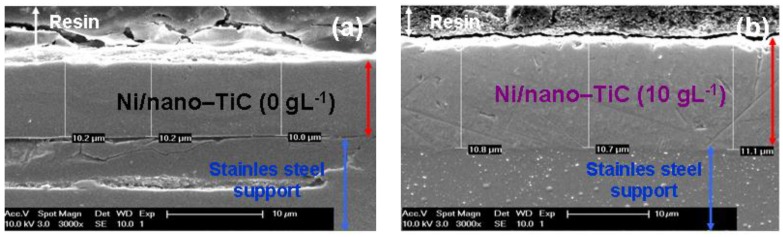

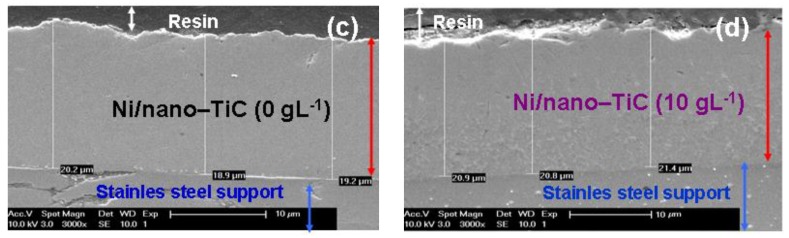
SEM micrographs of cross section for layer thickness measurement of pure Ni (**a**,**c**) and Ni/nano-TiC (10 g·L^−1^); (**b**,**d**) nanostructured composite layers obtained at a current density of 40 mA/cm^2^ and a codeposition time of: 15 min (**a**,**b**); and 30 min (**c**,**d**).

In order to confirm the layer thickness calculated by weighting, the real thicknesses were also analyzed by SEM on cross section of the samples (Figure 13). Figure 13 reveals the thicknesses measured by SEM in cross sections on for pure Ni layer and Ni/nano-TiC (10 g·L^−1^) obtained at a current density of 40 mA/cm^2^ and at codeposition time of 15 min. (Figure 13a,b) and at codeposition time of 30 min. (Figure 13c,d).

The coating thicknesses determined with SEM on cross section (Figure 13) are in full accordance with those calculated by weighting before and after electrodeposition and keep the same increasing trend with increasing concentration of TiC nanoparticles and time of electrodeposition. This increasing trend is achieved with all types of electrodeposited layers.

The scanning electron micrographs of electroplated layers performed in cross section confirm also a good adhesion of the layers to the stainless steel support.

### 3.6. Roughness of Electrodeposited Surfaces

**Figure 14:**
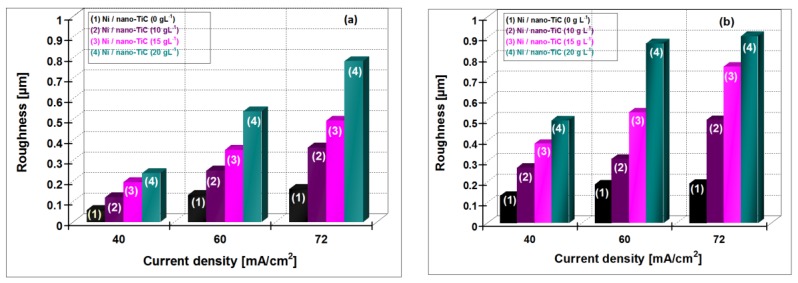
Roughness values as a result of 3D profiles for: Ni/nano-TiC (0 g·L^−1^) şi Ni/nano-TiC (10, 15, 20 g·L^−1^) produced under the same experimental conditions at deposition time of: (**a**) 15 min; (**b**) 30 min.

In Figure 14, the variation of the roughness for hybrid nanocomposite layers of Ni/nano-TiC (10, 15, 20 g·L^−1^) is shown compared with pure Ni layer obtained at current densities of 40 ,60 and 72 mA/cm^2^ at the electroplating times of 15 min and 30 min.

An increase of roughness with increasing current density and with the time of electroplating process for all the electroplated layers is observed. It is also evident that the roughnesses of Ni/nano-TiC hybrid nanocomposite layers are larger than pure nickel layers at all current densities applied.

Electrodeposition is considered a useful method to obtain superhydrophobic surfaces with special properies [19]. Superhydrophobicity of surfaces by electrodeposition could be achieved by: (a) surface roughness alone; (b) surface roughness and surface chemical modification with low surface energy material; and (c) by co-deposition of hydrophobic particles with a metal matrix [19].

The hybrid Ni/nano-TiC composite layers obtained revealed a higher roughness having included also TiC nanoparticles, which are considered as being hydrophobic; therefore, these layers are promising superhydrophobic surfaces for special application. Natural superhydrophobic surfaces, such as the lotus leaf or the legs of the water strider, have hierarchical nanoscale and microscale roughness [19]. The similarity with natural superhydrophobic surfaces is connected with roughness of the surface.

### 3.7. Nanoidentation of Surface Layers

The values of the nanoidentation results (indentation hardness (H), Vickers nanohardness (Hv), Elastic modulus (E) and H/E ratio) for the Ni/nano-TiC functional nanocomposite and the pure Ni coatings obtained under the same experimental conditions are indicated in Table 3 as a statistical average of three indentations.

Table 3 shows that the higher nanohardness values have been obtained for hybrid nanocomposite layers of Ni/nano-TiC (10 g·L^−1^), compared with that of the pure Ni layers. The higher hardness of Ni/nano-TiC composite coating in comparison with pure Ni layer can be accounted by the incorporation of hard TiC nanoparticles and in the Ni matrix, which limits the growth of the Ni grains. These results are in accordance with the observation on XRD analysis.

The hardness of Ni/nano-TiC hybrid composite layers obtained by electrodeposition is noticeably higher than those of pure nickel layers. This could be explained by the fact that TiC nanoparticles generate new locations of nucleation on the surface, resulting in grain refinement. Thus, the grain boundaries expand and hinder the motion of dislocations; as a result, hardness increases. This demonstrates once again that the Ni/nano-TiC hybrid nanocomposite layers have a higher hardness and could be more resistant to wear than the pure Ni layers.

## 4. Conclusions

The results confirm the incorporation of nano-TiC particles during the electro-codeposition process to obtain Ni/nano-TiC hybrid composite layers.

In order to obtain nanostructured hybrid composite layers with predefined thicknesses and good adhesion to support, the current density and time of electrodeposition are important parameters.

The TiC nanoparticle concentration in the nickel electrolyte influences their incorporation into the nickel matrix. An increase of the TiC wt. % nanoparticles’ codeposition into the Ni/TiC nanostructured layer is obtained by increasing both the concentration of TiC into the nickel plating electrolyte and by increasing the codeposition time.

By embedding the TiC nanoparticles into the Ni matrix, the morphologies of resulted hybrid nanocomposite layers of Ni/nano-TiC are changed and the effect is enhanced with increased concentration of nano-particles added to the nickel electrolyte. 

With the first addition of TiC nanoparticles (10 g·L^−1^) in the electrolytic bath, the borders/edges of the grains become blurred and the mean grain size is diminished compared to pure Ni coating at the same current density. Further increase in nanoparticle amount in the electrolytes leads to a characteristic cauliflower surface morphology of the Ni/TiC hybrid nanocomposite layer.

The current efficiency is higher during electro-codeposition of nano-TiC particles with nickel compared with pure nickel electrodeposition at the same current density due to embedding them into the nickel matrix and increasing the mass and volume of layer.

From XRD patterns of coatings, the inclusion of TiC nanoparticles into the nickel matrix by specific peaks was observed as well as its effects on crystal growth and structure of the nickel matrix and relative texture coefficients.

The presence of TiC nanoparticles leads to an increase in the intensity of the (200) peak and a decrease in intensity of (311) peak with further increase of TiC nanoparticles compared to the pure Ni layer. The preferred orientation of pure nickel layer is disturbed by nanoparticle inclusion to a non preferred one.

The addition of TiC nanoparticles into the bath modifies the preferred orientation of the pure Ni coating along the (311) crystallographic plane, reducing the RTC value (to about half), compared with that of the pure Ni coating. The TiC nanoparticle incorporation caused a loss of texture, indicated by the decreasing of RTC_311_ and the increasing of RTC_111_ and RTC_200_ values.

The presence of TiC nanoparticles into the nanocomposite coatings was also noticed during the XRD analysis. Adding TiC nanoparticles into the bath modifies the preferred orientation of the pure Ni coating along (311) the crystallographic plane, by reducing the RTC value to about half compared with that of the pure Ni coating.

The thickness of electroplated layers increases with increasing current densities; this upward trend was obtained for all types of codeposited layers.

The hybrid Ni/nano-TiC composite layers obtained revealed a higher roughness having also included TiC nanoparticles, which are considered as being hydrophobic; therefore, these layers are promising superhydrophobic surfaces for special application.

The Ni/nano-TiC hybrid nanocomposite layers have a higher hardness and could be more resistant to wear than the pure Ni layers.

## Figures and Tables

**Table 1:** Diffraction peak intensities of the pure nickel and Ni/nano-TiC functional composite coatings.

Crystalline Planes	Diffraction Peak Intensity (K-Counts)	
	Pure Ni Coating	Ni/Nano-TiC Coating (10 g·L^−1^)
(111)	18.34	9.88
(200)	5.29	3.81
(311)	13.29	3.38

**Table 2:** Relative texture coefficient *RTC_hkl_* of the pure nickel and Ni/nano-TiC functional composite coatings.

*RTC_hkl_*	Pure Ni Coating	Ni/Nano-TiC Coating (10 g·L^−1^)
*RTC*_111_	14.81	16.76
*RTC*_200_	10.17	15.40
*RTC*_311_	53.65	28.73

**Table 3:** Nanoidentation results as hardness, elastic modulus and H/E ratio for Ni/nano-TiC hybrid nanocomposite and pure Ni layers.

Type of Layer	Time of Deposition (min)	Identation Hardness (*H*) (GPa)	Vickers Hardness (Hv)	Elastic Modulus (*E*) (GPa)	H/E Ratio
Pure Ni layer	15	3.64	337.92	201.66	0.0180
Pure Ni layer	30	3.87	351.37	205.32	0.0188
Ni/nano-TiC layer (10 g·L^−1^)	15	4.87	451.37	221.64	0.0220
Ni/nano-TiC layer (10 g·L^−1^)	30	5.87	543.92	216.45	0.0271
